# Colony Stimulating Factor-1 and its Receptor in Gastrointestinal Malignant Tumors

**DOI:** 10.7150/jca.60379

**Published:** 2021-10-17

**Authors:** Hong-wu Li, Shi-lei Tang

**Affiliations:** General Surgery Department, The Fourth Affiliated Hospital of China Medical University, Shenyang, Liaoning Province, China, 110032.

**Keywords:** colony stimulating factor-1, colony stimulating factor-1 receptor, tumor-associated macrophages, gastrointestinal malignant tumors

## Abstract

Gastrointestinal malignant tumor is the fourth most common cancer in the world and the second cause of cancer death. Due to the susceptibility to lymphatic metastasis and liver metastasis, the prognosis of advanced tumor patients is still poor till now. With the development of tumor molecular biology, the tumor microenvironment and the cytokines, which are closely related to the proliferation, infiltration and metastasis, have become a research hotspot in life sciences. Colony stimulating factor-1 (CSF-1), a polypeptide chain cytokine, and its receptor CSF-1R are reported to play important roles in regulating tumor-associated macrophages in tumor microenvironment and participating in the occurrence and development in diversities of cancers. Targeted inhibition of the CSF-1/CSF-1R signal axis has broad application prospects in cancer immunotherapy. Here, we reviewed the biological characters of CSF-1/CSF-1R and their relationship with gastrointestinal malignancies.

## Introduction

Malignant tumors of the gastrointestinal (GI) tract are one of the common malignant tumors in China, among which gastric cancer is the fourth most common cancer in the world and the second cause of cancer death 1, 2. On account of a high frequent occurrence of lymphatic and liver metastasis, advanced GI malignant tumors lead to a poor prognosis and a serious threat to the life and living quality of patients 3-5**.** With the development of tumor molecular biology and related disciplines, tumor microenvironment and the involved cytokines, which are closely related to the occurrence, proliferation, infiltration and metastasis of GI malignant tumors, have become a research hotspot in life sciences 6. Understanding the tumor microenvironment (TME) and its role in tumor treatment is the key step in the development of more effective treatment methods 7. Colony stimulating factor-1 (CSF-1), a polypeptide chain cytokine, has been found that when combined with colony stimulating factor-1 receptor (CSF-1R), it could mobilize a variety of bone marrow precursor cells, promote cell proliferation, differentiation and migration, and enhance the function of mature granulocytes 8-10. CSF-1R is mainly expressed on the surface of hematopoietic stem cells, myeloid progenitor cells, and mature granulocytes 11, 12. Recent studies have found that CSF-1 in the tumor microenvironment could combine with its receptor CSF-1R in liver cancer, ureteral cancer, pancreatic cancer, gastric cancer, intestinal cancer and other malignant tumors 7, 13-17, and participate in biological processes such as promoting tumor cell proliferation, inhibiting tumor cell apoptosis and inducing blood vessel formation, etc. Targeted inhibition of the CSF-1/CSF-1R signal axis has broad application prospects in cancer immunotherapy 18-20. Therefore, it is important to explore the CSF-1 and CSF-1R signaling pathways related to tumor-associated macrophages (TAM) and their specific mechanisms in the comprehensive prevention and treatment of gastrointestinal malignancies. Here, we summarized the biological functions of CSF-1/CSF-1R and reviewed cancer promoting mechanisms regulated by CSF-1/CSF-1R axis in neoplasms, particularly in gastrointestinal tract malignant tumors.

## Characteristics, structure and function of CSF-1 and its receptor CSF-1R

### Characteristics, structure and function of CSF-1

CSF-1 is an important cytokine *in vivo* and a member of the hematopoietic growth factor family. It plays an important role in different stages of hematopoiesis 21. CSF-1 could directly act on CSF-1R, usually expressed in platelets, hematopoietic stem cells, bone marrow progenitor cells and mature bone marrow cells, leading to the acceleration of the formation of granulocyte colonies, the differentiation and proliferation of bone marrow cells, the enhancement of the function of mature neutrophils, and the acceleration of cell migration 22. In the study of hematopoietic cells *in vitro*, CSF-1 can stimulate different hematopoietic stem cells to form cells in semisolid media and stimulate bone marrow and spleen macrophage cell lines 23. In recent years, studies have shown that CSF-1 is highly expressed in many malignant tumors, which can accelerate invasion, proliferation and metastasis and induce the formation of tumor vessels by binding with CSF-1R 16, 19, 24. The high expression of CSF-1 can also be observed in tissues obtained from surgery, suggesting that high expression of CSF-1 may be an indicator of the prognosis of patients 25.

CSF-1 is a glycoprotein composed of 174 amino acid residues 26. The full length of the CSF-1 gene is 2.5 kb, including 5 exons, 4 introns, 5 cysteines, and two disulfide bonds, which are formed between Cys-36 and -42 and Cys-64 and -74. Disulfide bonds are an important factor in maintaining the biological function of CSF-1 27, 28. Naturally formed CSF-1 is linked by four single rings through amino acids. The CSF content in the healthy state is low. When inflammation occurs in the body, the concentration of CSF in plasma increases significantly 29, 30.

### General characteristics, structure and function of CSF-1R

CSF-1R belongs to the receptor tyrosine kinase signaling system. One of the characters of such a receptor is that when the ligand binds to the receptor recognition sites outside the cell membrane, the receptor will then aggregate and phosphorylate the tyrosine kinase-activated receptors within the cell membrane, finally leading to the phosphorylation of the tyrosine residues of effector proteins and the alteration of the biological activity of the effector 31. A recent study has demonstrated that in primary colorectal cancer, the elevated expression of receptor tyrosine kinase CSF1R at the tumor invasion was associated with poor patient survival and a mesenchymal-like subtype 24. Effector proteins include many factors related to cell proliferation and differentiation and many other components of signal-mediated systems. An obvious characteristic of this system is that in addition to rapid reaction time, it can also exert a long-term effect on cells 18. Since most factors that regulate cell proliferation and differentiation work in this way, thus this receptor is closely related to the occurrence and development of tumors.

CSF-1R, also known as FMS kinase, is the coding product of the c-fms proto-oncogene 32**.** C-fms is located at 5q33-3 of the human chromosome. Human CSF-1R synthesizes a 130 kDa immature transmembrane glycoprotein (gp130c-fms) from the rough endoplasmic reticulum, which is then modified by N-terminal oligosaccharide chains to form a mature 150 kDa glycoprotein (gp150c-fms) during intracellular transport. CSF-1R is secreted from the Golgi to the cytoplasm and then localized to the cell membrane. It can be converted from gp130c-fms to gp150c-fms within 1 hour 33. Human CSF-1R is a 972-amino acid polypeptide, consisting of four main parts: a 19 amino acid signal peptide sequence, a 493 amino acid ligand recognition and binding sequence outside membrane, a 25 amino acid transmembrane segment, and a 435 amino acid C-terminal tyrosine kinase catalytic sequence 34. CSF-1R is very similar in structure with platelet-derived growth factor (PDGF) receptor and belongs to the type III receptor tyrosine kinase family**.** The extracellular domain consists of five immunoglobulin-like rings for ligand binding. The intracellular domain contains tyrosine residues for self-phosphorylation, the kinase catalytic region contains three tyrosine self-phosphorylation sites (699,708,723), and the C-terminal region also has a tyrosine residue (809) for self-phosphorylation 35. After binding with CSF-1R, a ligand-receptor complex is formed, which is then endocytosed by cells and degraded in lysosomes. This is referred to as the internalization of CSF-1R 36. The extracellular signal produced by the binding of the receptor to its ligand is transmitted and amplified to the cells, which leads to the activation of tyrosine kinase and the phosphorylation of the receptor itself, thus initiating and stimulating the signal transduction pathway of cell proliferation. Therefore, the activation of the receptor tyrosine kinase is the first step for CSF-1R to play its role 37.

When membrane receptors are abnormally activated, tyrosine residues can be phosphorylated to induce ligand conformational changes that stimulate continuous activation of tyrosine kinases, leading to cell growth and proliferation and eventually carcinogenesis 38. Choudlhury et al. reported that an abnormal increase in the activity of the CSF-1R tyrosine kinase domain can activate the proto-oncogene c-raf-1, thus increasing the activity of serine/threonine kinases, which encode the product of CSF-1R. This can enhance or accelerate the transduction of intracellular growth stimuli, resulting in the amplification effect on the biological function of CSF-1R 39. Macrophage colony-stimulating factor binds to CSF-1R and activates its tyrosine kinase, which plays an important role in the survival, proliferation, differentiation and embryonic development of monocyte macrophage lines 13. Overexpression of CSF-1R and its ligands causes abnormal activation of the cell signaling pathway mediated by CSF-1R and participates in the processes of tumorigenesis and inflammation. Similar studies have shown that CSF-1R and its ligand CSF-1 can activate the receptor tyrosine kinase signaling system and continuously increase the activity of tyrosine kinase, thus changing the biological activity of the effector proteins. This allows the corresponding cells to continue growing and proliferating, which play a long-term role in the cell, and finally develop into cancer 16. Therefore, inhibitors targeting CSF-1R kinase can inhibit receptor phosphorylation and block the receptor-mediated cell signaling pathway, which is a potential target drug for the treatment of malignant tumors and inflammatory diseases 40.

## The relationship between CSF-1/CSF-1R axis and TAM in malignant tumors

Recent studies have shown that TAM is closely related to macrophage colony stimulating factor (M-CSF) 41. Macrophages that reside in the tumor microenvironment are called tumor-associated macrophages. They are the main inflammatory immune cells in the tumor microenvironment and are involved in tumor immunosuppression, angiogenesis, invasion, and metastasis 42, 43.

The interaction between CSF-1 and CSF-1R could cause receptor dimerization, tyrosine phosphorylation, and the subsequent interaction with multiple intracellular signaling pathways such as Ras, MAPK, PI-3K, JAK, finally producing various biological effects 44. CSF-1, as a tumor molecular marker, are highly expressed in a variety of tumors. Over-expression of CSF-1 or CSF-1R is associated with tumor aggressiveness and poor prognosis 45, 46. Recently, it has been found that the application of inhibitors can block the CSF-1R receptor and significantly reduce the invasiveness and proliferation of endometrial cancer, of which the progression could be promoted by TAM 47. CSF-1 secreted by endometrial cancer cells promotes the migration and proliferation of macrophages. The results show that the interaction between CSF-1 and its receptor plays an important role in promoting macrophage infiltration and endometrial cancer progression 48.

According to their phenotypes and secreted cytokines, TAM is divided into two types of polarization: classically activated M1 type and selectively activated M2 type macrophages 49, 50. M1 type macrophages mainly secrete pro-inflammatory factors and exert host immune functions against microbial inflammation and killing tumor cells. M2 type macrophages play a local anti-inflammatory effect in the later stage of inflammation, promote wound repair and fibrosis, participate in the formation of tumor stroma, and promote tumor growth, metastasis, and tumor angiogenesis 51. Macrophage polarization typing is the body's need for the diversity of immune function, which is related to the microenvironment and disease state of macrophages. Various pathological products or factors *in vivo* and *in vitro* become an important inducement for the macrophage polarization. Abnormally high expression of CSF-1 is related to pathological processes such as tumors and inflammation 52, 53. Due to the different functions of M1 and M2 macrophages, there may be mutual conversion between these two types of cells. Blocking the CSF-1/CSF-1R signaling pathway interferes with tumor progression by regulating TAM, reducing tumor invasion and proliferation 54.

Previous studies have demonstrated that CSF-1 and interleukin 34 (IL-34) signal via CSF-1R play important roles in macrophage differentiation in several inflammatory and oncological preclinical models 55, 56. For example, Blockade of both CSF-1 and IL-34 is protective in murine models of colitis and ileitis 55. However, numerous recent studies have found that CSF-1/CSF-1R axis blockade can improve the efficiency of immune checkpoint inhibitors, especially PD-L1. Thus, in terms of tumor control, the combination therapy targeting CSF-1/CSF-1R axis and immune checkpoint molecular has more reliable efficacy 57, 58. CSF-1/CSF-1R signaling mediates tumor-associated macrophages recruitment and M2 polarization. In experimental mesotheliomas, combined a highly selective small molecule CSF-1R inhibitor-BLZ945 with an anti-PDL1 agent was more effective in retarding tumor growth compared to each monotherapy 58. Moreover, AMG 820, an anti-CSF-1R antibody, showed acceptable safety profile in combination with pembrolizumab in adults with advanced solid tumors by reducing CSF-1 dependent CD16 expressing monocytes, and increasing PD-L1 expression and infiltrating T-lymphocyte numbers in advanced solid tumor biopsies 57.

**Figure 1:**
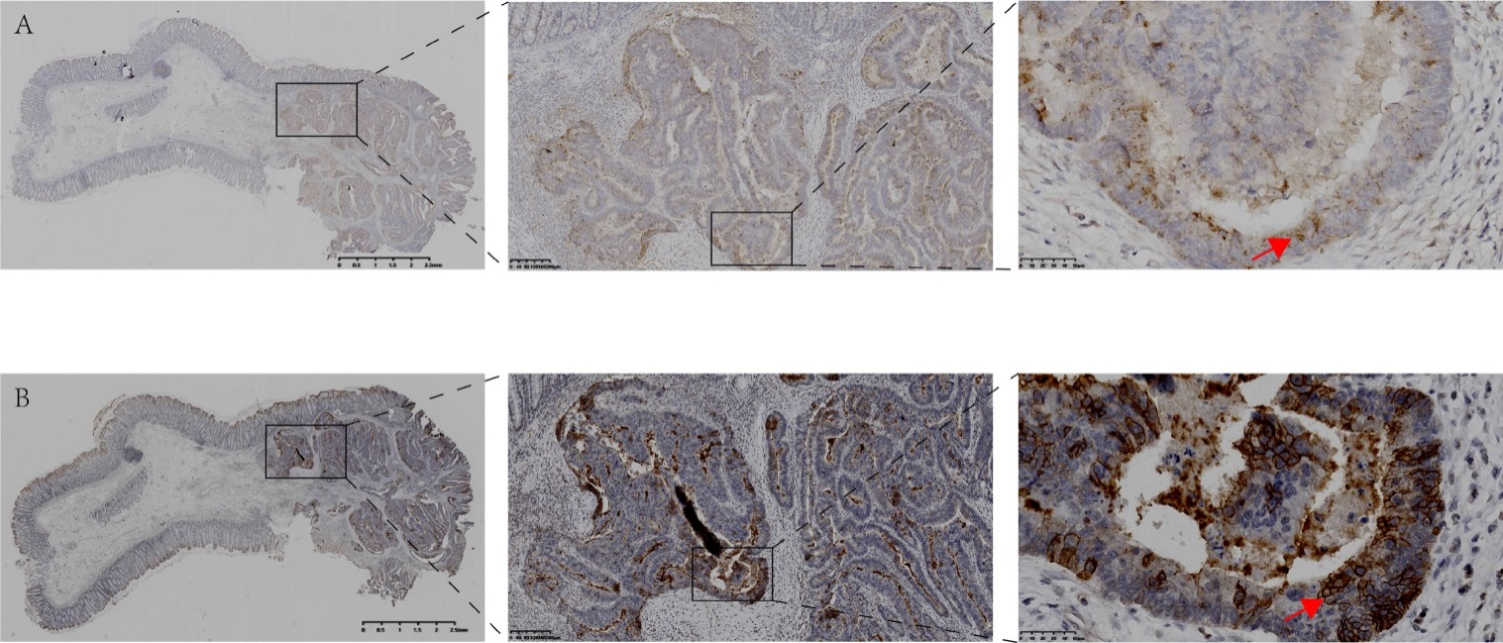
** Expression and localization of CSF-1 and CSF-1R in the same human colon tissue. A.** Immunohistochemical staining showed the expression and localization of CSF-1 in human colon cancer and para-cancerous tissues. **B.** Immunohistochemical staining demonstrated the expression and localization of CSF-1R in the same case. The expression of CSF-1 and CSF-1R in tumor tissue with abnormal structure was significantly higher than that in para-cancerous colon tissue with normal glandular structure. The expression level of CSF-1 in cancer cells is lower than that in tumor stroma, indicating TME may be the main sources of CSF1 (red arrow in Fig 1.A). CSF1-R is mainly expressed in tumor cell membrane (red arrow in Fig 1.B), which is different from the location of CSF-1.

Alternative ligands of CSF-1R, including IL-34 59, have been discovered, but most macrophages require signaling via the CSF1-CSF-1R axis. Except for macrophage, CSF-1/CSF-1R axis can promote tumor progression by interacting with other cells in tumor microenvironment, for instance, CAFs 60. In addition to fibroblasts, CSF-1 can be secreted by tumor cells, suggesting that it may play a pro-tumorigenic role (**Fig. 1**). Consistent with this, in metastatic PDAC, tumor-cell-derived CSF-1 induces macrophages to produce granulin, a secreted glycoprotein that promotes fibroblast activation and spurs tumor growth 61.

The CSF-1/CSF-1R signaling pathway modulates the production, differentiation, and function of TAMs. However, the discovery of selective CSF-1R inhibitors devoid of type III kinase activity has proven to be challenging in tumor treatment. Barbara Czako *et al*. discovered a potent, highly selective, and orally bioavailable CSF1R inhibitor, IACS-9439, which was proposed as a potential therapy to reduce TAMs, especially the protumor, immune-suppressive M2 TAMs and promote macrophage polarization toward the M1 phenotype by targeting CSF-1R 62. Recently, immunotherapy has gradually become the focus of cancer treatment. However, the majority of patients with “cold” tumors do not benefit from immunotherapy 63. Interestingly, a targeted delivery strategy, which modified cell-penetrating TAT peptide by using CSF-1R inhibitor, successfully activate immune response through blocking the CSF-1/CSF-1R pathway and reducing M2 macrophages and thus promoting anti-tumor effector CD8+T-lymphocyte infiltration in “cold” colon cancer 64.

## Expression and significance of CSF-1 and CSF-1R in gastrointestinal malignant tumors

CSF-1 is a hematopoietic growth factor that acts through the cfms/CSF-1R. The CSF-1/CSF-1R axis is considered to be involved in the invasion and development of various types of cancer 65, 66. Studies have reported that elevated expression of CSF‐1/CSF‐1R significantly correlated with disease progression and with a poor overall survival (OS) and disease-free survival (DFS) of patients with gastric cancer. Furthermore, the high co-expression of CSF-1 and CSF-1R was an independent prognostic factor for OS, DFS, lymph node and peritoneal metastasis, indicating that the CSF-1/CSF-1R axis may be a clinically useful prognostic and predictive biomarker for lymph node and peritoneal metastasis and a potential therapeutic target in gastric cancer 16. Other studies have found that CSF is a key factor to drive CXCL8 secretion in M2 type TAM. The high expression of CXCL8 is significantly associated with decreased CD8^+^ and Ki67^+^ T cells infiltration and unfavorable clinical outcome in gastric cancer patients. Importantly, the authors provided evidence that tumor-associated macrophages-derived CXCL8 determines immune evasion through autonomous PD-L1 expression in gastric cancer, suggesting that it may be a promising strategy to block the CXCL8 pathway to increase anti-tumor immunity in gastric cancer 29. The CSF-1/CSF-1R axis may be a biomarker for the clinical diagnosis of lymph node and peritoneal metastasis of gastric cancer and is a potential therapeutic target for gastric cancer^16^. Recombinant human granulocyte colony-stimulating factor (rhGCSF) has been widely used in the treatment of granulocytopenia induced by chemoradiotherapy. *In vitro* studies have shown that CSF can also be produced by tumor cells and stromal cells, and it could promote tumor fitness and cell proliferation and metastasis 67. Patients with high CSF expression have a rapid disease progression and short survival time. Inhibition of CSF can reduce tumor angiogenesis and inhibit tumor growth. Fan et al. 68 studied the value of serum CSF in the diagnosis and prognosis of gastric cancer. It was found that the serum CSF level in gastric cancer patients was significantly higher than that in healthy patients. With advanced TNM stage, the serum level of CSF gradually decreased. High expression of CSF in gastric cancer tissues was significantly increased, which was positively correlated with TNM stage and lymph node metastasis 68, 69. Morris et al 70 studied the expression and function of G-CSF and G-CSFR in gastrointestinal cancer tissues and cells. It was found that CSFR was highly expressed in 90% of gastrointestinal cancer. Production of CSF was increased in interstitial myofibroblasts and cancer cells, and the proliferation and migration of cancer cells expressing CSF were enhanced. These processes depend on phosphorylation of the ERK1/RSK signaling pathway 70. Scholars have found that CXCL8 is a key chemokine for gastric cancer metastasis, which is mainly derived from TAM. In gastric cancer, through inducing macrophages and PD-L1 to participate in the immunosuppression of the tumor microenvironment, CXCL8 inhibitors could trigger the anti-tumor response, which could provide potential therapeutic effects for gastric cancer patients 29. Another research team studied the CSF3/CSF3R signaling in colon and rectal cancers, and results indicated that CSF3/CSF3R expression was correlated with changes in T cell and macrophage signatures and also correlated with genes that are associated with poor colorectal cancer prognosis 71.

Recent works showed that serum CSF-1 has great value in the diagnosis and progression of colorectal cancer (CRC), making it an independent prognostic factor for the survival of patients with CRC 72. As mentioned before, increased CSF-1R, CSF-1 and IL-34 expression in primary CRC was associated with a mesenchymal-like subtype and tumor invasion as well as distant metastasis 24, 72. Recently, immunotherapy has gradually become the focus of cancer treatment. But most patients with “cold” tumors do not benefit from immunotherapy 63. Interestingly, a targeted delivery strategy, which modified cell-penetrating TAT peptide by using CSF-1R inhibitor, successfully activate immune response through blocking the CSF-1/CSF-1R pathway and reducing M2 macrophages and thus promoting antitumor effector CD8^+^T-lymphocyte infiltration in “cold” colon cancer 64.

It has been found that abnormal expression of CSF-1/CSF-1R axis can also occur in malignant meningiomas, hepatocellular carcinoma, and pancreatic cancer, where CSF-1/CSF-1R blockade reprograms tumor-infiltrating macrophages and improves response improves response to T cell checkpoint immunotherapy 66, 73, 74.

**Figure 2:**
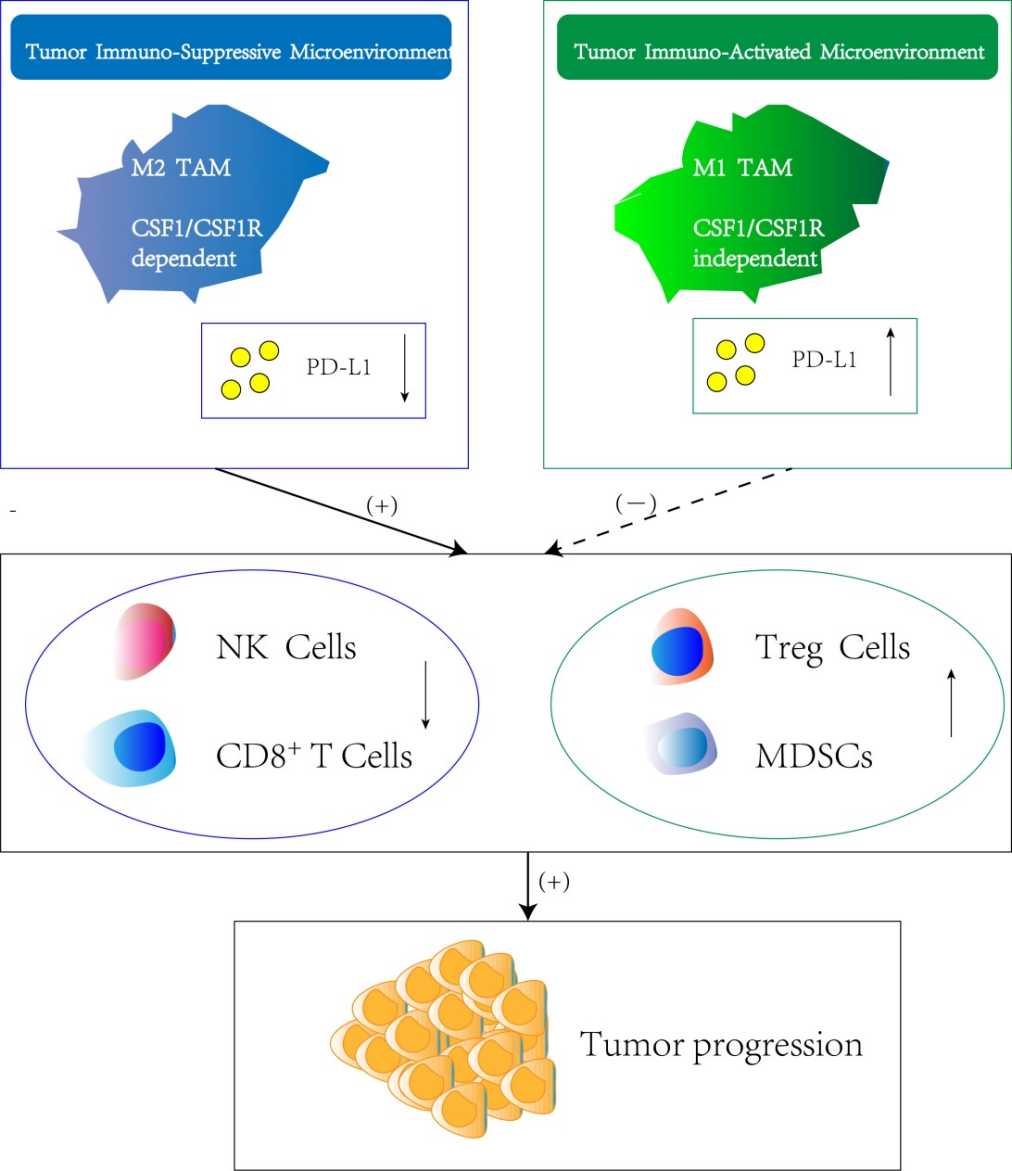
** Regulation of immune suppression or activation by TAM subtypes.** Macrophage polarization within the tumor immune-suppressive microenvironment is highly dependent on CSF-1/CSF-1R axis which originates either from tumor cells or stromal cells. The M2 TAM phenotype induce the downregulation of PD-L1 in TME, which result in silencing of immune effector cells such as nature killing cells and CD8^+^ T cells. Meanwhile, the infiltration and function of other immune-suppressive cell types such as T regulatory cells (Treg cells) and Myeloid-derived suppressor cells (MDSCs) is stimulated, thus promoting tumor progression. In contrast, M1 TAM are attributed with tumoricidal functions showing the opposite effect of M2 TAM.

The tumor microenvironment includes the structure, function and metabolism of the tissues in which tumors are located, as well as the external environment in which tumors themselves and surrounding immune cells and immunoregulatory factors are formed 75. The occurrence, growth and metastasis of tumors are closely related to the microenvironment. CSF can recruit neutrophils, monocytes and macrophages, etc. to the adjacent areas of the tumors, promoting the development of tumors. Li et al. found that the infiltration of bone marrow-derived suppressor cells and macrophages in a mouse colorectal cancer model was related to the increase of CSF. CSF could mobilize bone marrow-derived suppressor cells to migrate from bone marrow to the colon, promoting the proliferation of bone marrow-derived suppressor cells and inhibiting their apoptosis and then inducing the occurrence and development of colon cancer 76. Studies have reported 77 that TAM in GI tumor microenvironment, responding to stimuli such as growth factors and cytokines, could be polarized into a state with pro-tumor activity or anti-tumor activity (M1 or M2). CSF is a cytokine that can affect immune cells in the tumor microenvironment and has tumor-promoting activity (**Fig. 2**). A study investigated the effect of CSF/CSF-R on the progression of colon cancer and pancreatic cancer in a mouse model and the results showed that in the absence of CSF-R, macrophage-related tumor cytotoxicity was amplified, indicating that CSF/CSF-R is an important clinical application target for controlling tumor microenvironment and gastrointestinal tumor progression 72, 78.

## Signaling pathway regulation mechanisms involved by CSF-1 and CSF-1R

**Figure 3:**
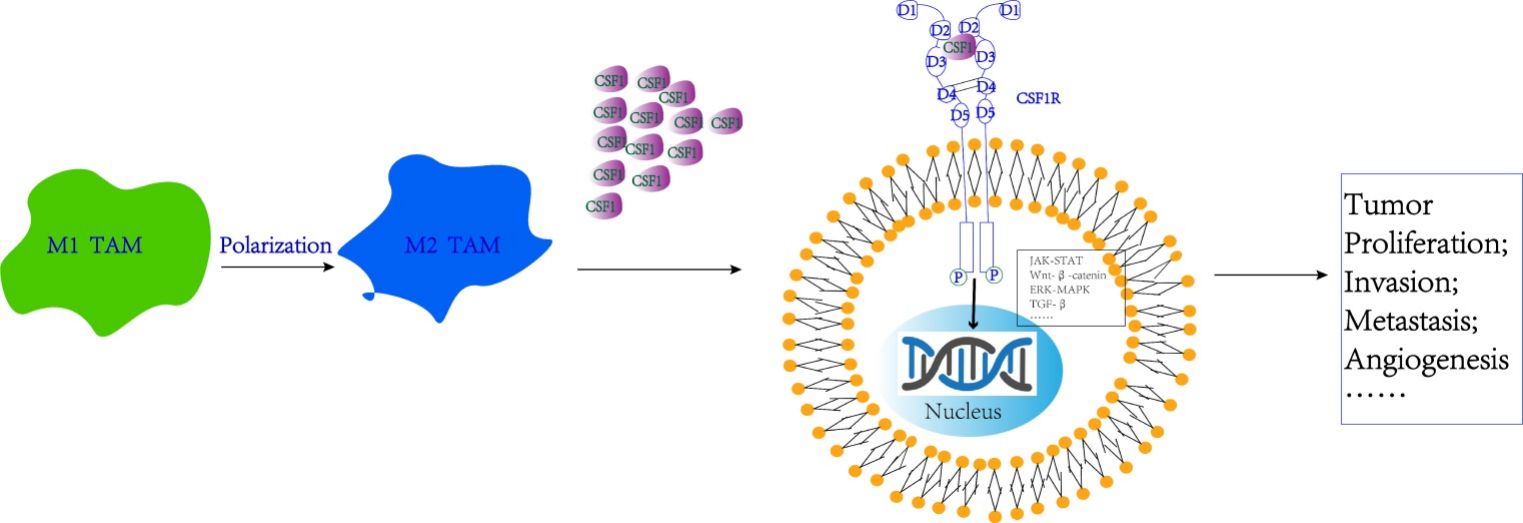
Signaling pathway regulation mechanisms involved by CSF-1 and CSF-1R. CSF-1R binding to the CSF-1 ligand on the surface of cancer cells can activate multiple intracellular signaling pathways, including JAK-STAT, Wnt, MAPK, TGF-β signaling pathway, thus promoting tumor proliferation, invasion, metastasis, and angiogenesis.

CSF-1R binding to the CSF-1 ligand on the surface of cancer cells can activate multiple intracellular signaling pathways to promote proliferation, invasion, metastasis, and angiogenesis 79. Blocking CSF-1R with antibodies can reduce the activation of CSF-1 and prevent the proliferation and metastasis of malignant tumors. It was found that CSF could participate in the activation of the JAK tyrosine kinase pathway 80, the Wnt3a pathway 81, the PI3-kinase pathway 82, and the ERK1/2 pathway 83 (**Fig. 3**). JAK/STAT is generally believed to be the main pathway for CSF signal transmission 80. After CSF binds to its receptor, JNK is activated, which could phosphorylate tyrosine residues in the intracellular segment of CSFR and then re-localize the STAT family proteins into the nucleus, binding to the promoter region of the target gene and inducing effector protein expression 84. Recent work has demonstrated that the elevated expression of receptor tyrosine kinase CSF-1R act as a direct miR-34a target and as a negative effector of p53/miR-34a axis, promoting progression of colorectal cancer by activating STAT3 pathway 24. Notably, CSF-1R does not necessarily receive the stimulation of CSF-1. For example, by triggering receptor expressed on myoid cells-2 (TREM2), CSF-1R is a high risk marker for Alzheimer's disease, which explains the disadvantage of mono targeting CSF-1R from another point of view 85.

Understanding the signaling mechanism of CSF-1 and CSF-1R in cancer and other diseases and taking appropriate measures to block CSF-1/CSF-1R signaling is a promising new immunotherapy with potential for future clinical application. Studies have found that CSF-1 is one of the most common proinflammatory cytokines, and it can cause various inflammatory diseases 65. It plays an important role in the development and progression of osteoarthritis, cancer and other autoimmune diseases. CSF-1 plays a role by binding CSF-1R, causing a cascade reaction of signaling pathways leading to cell proliferation and differentiation, promoting the differentiation and survival of monocytes, macrophages and osteoclasts. CSF-1R is overexpressed in many cancers and cancer-related macrophages and is therefore used as a drug target for cancer and inflammatory diseases. Some CSF-1R inhibitors have been successfully applied to these diseases.

## Summary

CSF is widely used in clinic to treat granulocytopenia patients after chemotherapy. Increasing research has shown that CSF-1 and CSF-1R are expressed in malignant tumors of the digestive tract and can promote the growth, migration and invasion of tumors**.** CSF-1 and CSF-1R play a role through multiple signaling pathways by inhibiting the immune response of the body. Blocking the CSF-1/CSF-1R axis can not only directly treat tumor cells with CSF1R, as a targeted therapy, but also can promote the polarization of TAM and improve the tumor microenvironment, thereby exerting an anti-tumor effect**.** However, the mechanism of CSF-1 and CSF-1R in GI malignant tumors is still not clear, and the mechanism of how inflammatory immune cells participate in the occurrence and development of malignant tumors is not perfectly defined. Therefore, more in-depth exploration and clinical validation are needed to improve the effects of targeted drugs and comprehensive treatments and to help identify new markers of malignant tumors for early diagnosis and evaluation of prognosis.
